# Editorial: Biological rhythms: Evolution, population biology, and adaptation

**DOI:** 10.3389/fphys.2023.1157631

**Published:** 2023-02-14

**Authors:** Charalambos P. Kyriacou, Pamela Menegazzi, David Dolezel

**Affiliations:** ^1^ Department Genetics and Genome Biology, University of Leicester, Leicester, United Kingdom; ^2^ Neurobiology and Genetics, University of Würzburg, Würzburg, Germany; ^3^ The Czech Academy of Sciences, AVCR, Institute of Entomology, Ceske Budejovice, Czechia

**Keywords:** circadian, adaptation, evolution, population, ecology, comparative

Our initial idea for this Research Topic was to attract articles with more ecological and evolutionary perspectives including papers that would tackle the thorny problem of the clock and Darwinian fitness. In addition, comparative studies on more exotic species with interesting ecologies would also be welcomed. Somewhat shamelessly, these subjects have been central to the research interests of the three editors for many years.

While it might appear obvious that having a circadian clock is essential for fitness, the fact that arrhythmic *Drosophila melanogaster per* ^*0*^ flies seem perfectly viable seems at odds with the importance one might attach to biological rhythmicity. Different approaches have been taken to assess the fitness benefits of having a circadian clock. The direct approach is to compete different clock variants against each other under defined environments and examine their Darwinian fitness. This type of study can most readily be performed with organisms with a very rapid generation time such as the photosynthetic cyanobacteria. In a classic study, *kaiC* period mutants in *Synechococcus* outcompeted the wild-type when the environmental light-dark (LD) cycle was set to resonate with the mutants’ endogenous period (Ouyang et al., 1998). This outstanding study remains the best example for demonstrating a direct association between circadian period and fitness.

The second approach has been to use neutrality tests developed in the late 1980s and early 1990s by population geneticists and apply them to natural clock gene variation such as *Drosophila period* and *timeless*. For *period*, the centrally encoded Thr-Gly repeat was observed to evolve under **balancing selection** in *D. melanogaster* and *Drosophila simulans* (Rosato et al., 1994; Rosato et al., 1996; Rosato et al., 1997). Follow-up experimentation using various Thr-Gly transgenes revealed that temperature compensation could be altered by manipulating this region (Sawyer et al., 1997; Peixoto et al., 1998). Furthermore, genetic variation in this repetitive region was latitudinally structured across two continents and was consistent with its role in maintaining a ∼24 h behavioural cycle under different thermal challenges (Costa et al., 1992; Sawyer et al., 2006). For *timeless,* the signature of **directional selection** was observed for a 5′ region that encoded a recent mutation that was spreading across Europe (Tauber et al., 2007). This mutation created a novel protein isoform of TIM, called LS-TIM*,* which altered circadian light sensitivity, diapause inducibility (Sandrelli et al., 2007; Tauber et al., 2007), and maintained rhythmicity in ‘white nights’, high latitude conditions of constant light but cycling temperatures (Lamaze et al., 2022). These three characters are expected to be differentially important at different latitudes and indeed the novel *ls-tim* mutation (and conversely, the ancestral *s-tim* allele) revealed spatially structured distributions in both Europe and N. America that were consistent with directional selection and the geographical origin and demography of the new allele (Tauber et al., 2007; Zonato et al., 2018).

Comparative studies are the substrate for evolutionary analyses and play a role in understanding why different species may show different expression patterns for clock genes. For example, the expression patterns of cryptochrome (CRY), the dedicated circadian photoreceptor and Pigment dispersing factor (PDF), the neuropeptide that transmits circadian information among clock neurons, are very different in high latitude *Drosophila virilis* group fly species compared to those that originate in the tropics, such as *D. melanogaster* (Menegazzi et al., 2017). These differences in neuronal expression appear to correlate with the apparently adaptive locomotor behaviour of the flies under the very long days during northern latitude summers. Manipulating the expression of CRY and PDF in different clock neurons of *D. melanogaster* partially mimics these neuronal patterns in the high latitude species. Furthermore, the locomotor behaviour of these *D. melanogaster* ‘mimics’ was similar to those of the *D. virilis* group species (Menegazzi et al., 2017). Consequently, changes in clock gene expression, not just sequence, can determine putative circadian adaptations to different environments.

However, tempting as it may be, we should not assume that changes in locomotor behaviour that would appear to be adaptive, reflect a measure of fitness. This is particularly so given that rhythmic behaviour measured in the wild in rodents and insects can be very different from that observed in the laboratory (Gattermann et al., 2008; Daan et al., 2011; Vanin et al., 2012). In this context, within our Research Topic, Andreatta et al. explore the life-history implications of the two *Drosophila tim* alleles mentioned earlier. *ls-tim* circadian photoresponses and diapause favour it in any seasonal environment (such as Europe) so eventually, this novel beneficial mutation may eliminate *s-tim.* Andreatta et al. reveal phenotypes that may adapt *s-tim* to warmer regions and could explain why the disappearance of the *s-tim* allele from southern Europe may be delayed. Pegoraro et al. describe a comprehensive analysis of genetic variation within the fly *cry* gene from natural populations that is not consistent with neutrality (Pegoraro et al..) Haplotypes show different circadian phenotypes, but surprisingly, responses to light are not affected. The study by Dani and Sheeba, heroically raises flies for 150 generations outside in tropical semi-natural conditions in India and compares the flies’ circadian eclosion phenotypes to matched controls reared in the gentler conditions of the laboratory (Dani and Sheeba). The results reveal dramatic adaptive changes in the phenotype and illuminate the evolution of this character under stressful natural conditions.

Moving away from *Drosophila*, in a sophisticated study of temporal synchronisation in honeybees, Siehler et al. observe that substrate borne vibrational plus volatile cues are important for social synchrony between individuals and reveals how evolution has fine-tuned these highly sensitive modality to impart time information to the hive (Siehler et al.). The final primary research paper by Thiel et al. is a seasonal study of Scandinavian brown bears, and for biological rhythm research it cannot get more exotic or dangerous (Thiel et al.). The changes in diel and infradian rhythms on physiology of these magnificent creatures is explored between their active stages and their hibernation phases.

In addition, we have three review papers. Rock et al. review the field of lunar-mediated cycles, including circatidal, semilunar and circalunar that are observed in most coastal organisms (Rock et al.). Lepidoptera have emerged as circadian model behaviour and for the related phenotype of compass-based navigation (Reppert et al., 2010). Brady et al., review circadian clocks in lepidoptera and focus on emerging pest control technologies (Brady et al.). Finally, Jabbur et al. review the clocks of Cyanobacteria, these primitive but spectacularly important organisms that contributed to the great oxygenation event, three billion years ago (Jabbur and Johnson). The authors juxtapose the past putative evolution of the *Kai* clock components with the extant model we observe now, and then discuss what climate change will mean to the evolution of the cyanobacterial clock as the Earth warms up.

In summary, this Research Topic of papers attempts to direct attention away from the more common hardcore molecular, ‘omic and neurogenetic approach to clocks and we hope that it will stimulate a wider appreciation of the ecological and evolutionary pressures that shape the expression of circadian systems.
